# Assessment of weighted topological overlap (wTO) to improve fidelity of gene co-expression networks

**DOI:** 10.1186/s12859-019-2596-9

**Published:** 2019-01-28

**Authors:** André Voigt, Eivind Almaas

**Affiliations:** 1 Network Systems Biology Group, Department of Biotechnology, NTNU - Norwegian University of Science and Technology, Trondheim, Norway; 2 K.G. Jebsen Center for Genetic Epidemiology, Department of Public Health and General Practice, NTNU - Norwegian University of Science and Technology, Trondheim, Norway

**Keywords:** Gene co-expression network, Weighted topological overlap, Correlation network, Biological network analysis

## Abstract

**Background:**

For more than a decade, gene expression data sets have been used as basis for the construction of co-expression networks used in systems biology investigations, leading to many important discoveries in a wide range of subjects spanning human disease to evolution and the development of organisms. A commonly encountered challenge in such investigations is first that of detecting, then subsequently removing, spurious correlations (i.e. links) in these networks. While access to a large number of measurements per gene would reduce this problem, often only a small number of measurements are available. The weighted Topological Overlap (wTO) measure, which incorporates information from the shared network-neighborhood of a given gene-pair into a single score, is a metric that is frequently used with the implicit expectation of producing higher-quality networks. However, the actual extent to which wTO improves on the accuracy of a co-expression analysis has not been quantified.

**Results:**

Here, we used a large-sample biological data set containing 338 gene-expression measurements per gene as a reference system. From these data, we generated ensembles consisting of 10, 20 and 50 randomly selected measurements to emulate low-quality data sets, finding that the wTO measure consistently generates more robust scores than what results from simple correlation calculations. Furthermore, for the data sets consisting of only 10 and 20 samples per gene, we find that wTO serves as a better predictor of the correlation scores generated from the full data set. However, we find that using wTO as a score for network building substantially alters several topographical aspects of the resulting networks, with no conclusive evidence that the resulting structure is more accurate. Importantly, we find that the much used approach of applying a soft-threshold modifier to link weights prior to computing the wTO substantially decreases the robustness of the resulting wTO network, but increases the predictive power of wTO networks with regards to the reference correlation (soft threshold) network, particularly as the size of the data sets increases.

**Conclusion:**

Our analysis demonstrates that, in agreement with previous assumptions, the wTO approach is capable of significantly improving the fidelity of co-expression networks, and that this effect is especially evident for cases of low-sample number gene-expression data sets.

**Electronic supplementary material:**

The online version of this article (10.1186/s12859-019-2596-9) contains supplementary material, which is available to authorized users.

## Background

In recent years, the system-level analysis of gene co-expression data sets as networks has become a much used approach to investigate and understand the rapidly increasing amount of available gene expression data. Such network analyses have generated important insights, including the identification of gene clusters involved in autism [1], different types of pancreatic cancer[2], seasonal differences in the human immune system[3], cardiac [4] and neural [5, 6] development, as well as evolution [7].

Many different methods have been developed for these network analyses, typically focused on generating networks where the genes are represented as nodes and a link between a pair of nodes corresponds to a measure of pair-wise gene expression profile similarities [8]. A variety of methods exist to attempt to quantify these similarities. The most common common approach, perhaps, is to determine the strength of a given link as a function of a correlation score (such as Pearson, Spearman, or similar) for the gene expression profiles, but other methods are also in use, for instance revolving around information theoretical approaches or Bayesian networks and directed acyclic graphs, which aim to filter out the strongest predictors of regulatory mechanisms [9]. As determining the best Bayesian network is an NP-hard problem, such methods depend on efficient heuristics, such as Markov Chain Monte Carlo [10] and steepest hill climbing [11] or sparsest permutation approaches [12]. From here on out, we focus on the correlation-based approach, and we will refer to the original and unmodified correlation between expression profiles as the ’base correlation’.

The nature of the correlation matrix approach, that of generating a score for all possible pairings of genes in the set, brings up a significant challenge as typical gene expression data sets contain simultaneous measurements of thousands of genes. Consequently, a complete pairwise comparison may involve computing correlations for millions of gene pairs, bringing up the issue of the statistical significance associated with a correlation score due to necessary *P*-value adjustments due to multiple testing: when such a large number of comparisons are made, spurious strong correlations are bound to occur in some number. This is of particular concern when dealing with data sets containing only a small number of measurements for each gene.

While large-scale expression data with measurements reported on hundreds of individuals are currently available [13–15], there are still many cases where the number of available samples are restricted to a dozen measurements per gene or even fewer. This is not unusual when studying medium to large non-human organisms (for instance, primates) or rare diseases, where the expense of maintaining the animals is prohibitive or there is a notable difficulty in identifying cases. Under such restrictions, even perfect or near-perfect correlations are not necessarily indicative of actual co-regulatory mechanisms.

A possible approach for dealing with the challenges emerging from small sample sizes, is that of using a network approach to look for information in the neighborhood of a gene pair [16, 17]- i.e., third-party genes that also connect to that gene pair. One such method is based on computing a score known as the weighted Topological Overlap (wTO) [18]. While wTO is a metric with established use, in particular by the popular R-package of WGCNA [19], its original stated purpose is primarily to identify modules. Additionally, according to the wording in the original article, the inclusion of topological overlap in WGCNA seems to be grounded more in previous use in a variety of network topics rather than an assessment with respect to gene expression data in specific. To our knowledge there has not been any study which aims to investigate the extent to which wTO is an improvement on base correlations when attempting to determine individual co-expressed gene-pairs in low-sample situations.

In this study, we have investigated the merit of wTO to improve on co-expression networks by first computing high-confidence networks from large data sets, and subsequently evaluating whether the use of wTO increases the predictive power of low-quality data sets artificially created from the same source.

## Methods

In order to perform our tests, we downloaded expression data from the GTEx consortium [13, 15] (http://www.gtexportal.org). We chose the “Whole blood” data set (with data for 23,152 genes), as it contains a very large amount of measurements per gene (338), which allows us to use the full data set as a reasonably accurate reference point. As an auxiliary test set for further confirmation, we also downloaded a separate data set obtained from mouse brains from the Gene Expression Omnibus [20], with the identifier GSE26500 [21]. This set was chosen to serve as an independent test compared to our human set, while exhibiting a comparable number of genes (25,698) and a reasonably large number of samples (198) in order to evaluate the reference co-expression.

Based on these gene-expression data sets, we simulate three levels of low-quality data by sampling nested sets of 10, 20 or 50 measurements (which we hereby refer to as points). The sampling was performed by randomizing the order of samples in the data sets, after which we simply picked the first 10, 20, or 50 points (according to randomized order) for each gene, so that all 10 points in the lowest-quality set are also contained in the 20-point set, which in turn is entirely contained in the 50-point set. The rationale for this nested sampling approach is to evaluate the performance of wTO on a sliding scale of increasing data quality, in order to identify a potential break-even point at which it either begins or ceases to outperform the base correlation. From here on out, we use the term “quality” specifically to denote the number of points in a set.

For a given set of *N* genes, the computation time of wTO is longer than that of a simple correlation analysis; *O*(*N*^3^) as opposed to *O*(*N*^2^). Consequently, in order to reduce running times of our investigation to a more manageable duration, we reduce the number of genes in the simulated low-quality sets (and corresponding reference sets) to 1000 genes (out of the 23,152 available) in each set. This reduction in gene-set size also allows us to create an ensemble of 20 separate groups (without any shared genes between them) of nested low-quality sets, with corresponding reference sets, in order to account for potential variability in the predictive performance of the low-quality sets. In a procedure somewhat similar to the nested sampling approach for reducing the number of data points per gene, we generated the 20 1000-gene data sets by randomizing the order of genes in our original set, and dividing it into 20 consecutive sets (the remaining 3152 genes being omitted from the study). This process is effectively identical to a random draw of 20 separate 1000-gene sets without replacement.

While a variety of different metrics for calculating pairwise co-expressions as a starting point for a network analysis are currently in use, we have focused our efforts on testing two commonly used types: Spearman correlation and biweight midcorrelation (bicor) [22–24].The latter has been argued to generate more robust similarity measures for gene co-expression networks [24]. As a further test, we also evaluate the effect of applying soft thresholding to the bicor metric, where the bicor value is raised to a pre-defined exponent in this case chosen to be 6, to accentuate strong correlations and filter out weaker ones. For each metric, we compute the base co-expression measure and the corresponding wTO for the simulated low-quality sets and the reference sets, defined as follows for a gene pair (*i*,*j*):

1 $$ wTO(i, j) = \frac{w_{ij} + {\sum\nolimits}_{k\neq i, j}{w_{ik} w_{kj}}}{\min\left({\sum\nolimits}_{k} w_{ik}, {\sum\nolimits}_{k} w_{jk}\right) + 1 - w_{ij}},   $$

where *w*_*ab*_ denotes the absolute value of the correlation score between genes *a* and *b*.

We then evaluate the performance of the measures on low-quality sets against the reference set, using two rank-based tests: (A) Jaccard index for the top 1000 pairs in each low-quality set as compared to the reference sets, and (B) Spearman correlation between the co-expression/wTO scores in the low-quality sets and the reference sets.

Our motivation for the choice of rank-based tests is as follows: For a given threshold, the proportion of false positives within a given selection is essentially determined by the proportion of factually uncorrelated genes above a given rank in the data set. Any perturbation (e.g. due to differences in methodology or sample size) of the co-expression matrix that does not alter the order of pairs could, in principle, be counteracted by an appropriate change in the cut-off, yielding the desired reference network. On the other hand, if spuriously correlated (low-rank in the reference set) gene-pairs suddenly exhibit larger edge weights (i.e., higher rank in the low-quality set) than the factually correlated genes, false positives become inevitable. Identifying changes in ranking of pairs between sets is therefore critical. Our intention with the Spearman test is to identify this across the entire gene set, while the purpose of the Jaccard test is to focus on the most strongly co-expressed pairs, which are usually of main interest for a biological study.

In order to test the impact of wTO usage on network structure, we generated unweighted networks from each of the complete matrices using the 1000-gene sets for all of the chosen data quality levels by retaining all edges above a given cut-off. Determining an appropriate cut-off threshold for building networks from correlation matrices is a major field of gene co-expression analysis in particular and network science in general, and a variety of criteria have been proposed for this purpose. One might, for instance, set it to correspond to a desired *p*-value under the null hypothesis that the data is independent. Other proposed methods specifically suggesting bicor, Spearman or Pearson correlations as the co-expression metric involve choosing a cut-off so that the network follows a scale-free topology [19], or setting the cut-off near abrupt transitions in the nearest neighbour spacing distribution [25]. A detailed investigation in the choice of cut-off and impact thereof is, however, outside the scope of this paper. To build networks from our complete correlation and wTO matrix, we therefore opted for the straightforward approach of selecting the top 0.2% of gene pairs (from the 500,000 pairs per set). Community detection on these networks was conducted using the Louvain algorithm [26].

## Software

Computation of base correlation matrices (both Spearman and bicor) was conducted using in-house software written in C ++. Soft-thresholded correlation and wTO matrices were computed from the base correlation matrices using in-house software written in Python. Performance evaluation was conducted in Python using the libraries scipy [27] and NetworkX [28]. All box plots were generated in Python, using matplotlib [29]. The code used has been made available on GitHub (https://github.com/andrevo/DiffCoEx-WTO).

## Results

### Accuracy of pair interactions

**Fig. 1:**
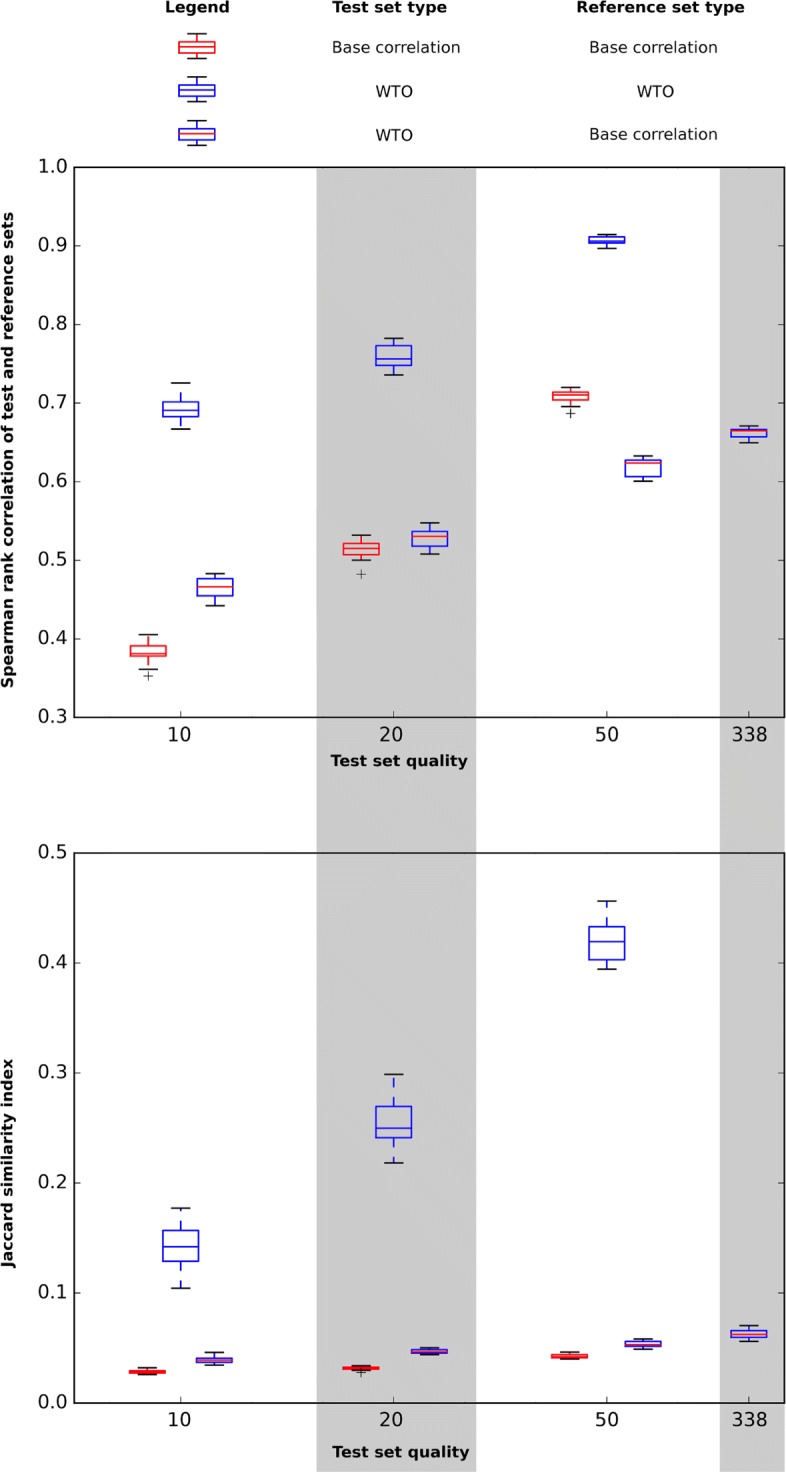
Comparison of fidelity to full-sample data between non-modified pairwise correlations (biweight midcorrelation) and wTO of the bicor network, according to two tests: Spearman rank correlation of edge pairs and Jaccard similarity of the top 1000 edge pairs

We assess the predictive power of a low-quality set with respect to the complete high-quality human whole blood set by computing the Spearman correlation between the low-quality and high-quality link scores. Figure 1 shows the predictive power of base gene co-expression correlation and the wTO computed from lower-quality data sets using the 20 ensembles. We clearly see that for all reduced quality levels, the low-quality wTO predicts the reference wTO significantly better than the low-quality correlation (using bicor) is able to predict the reference correlation. We observe similar results in the mouse data set (Additional file 1). In other terms: wTO networks are substantially less sensitive to small sample sizes (in terms of measurements per gene) than a network based on simple pairwise correlations. We also find that the choice of Spearman or biweight midcorrelation as the base co-expression measure has minimal effect on performance fidelity (see Additional file 2: Performance test of wTO against base Spearman).

However, wTO is not a direct measure of co-expression, and we ask ourselves: if one is only interested in actually quantifying the direct relationship between the expression levels of two genes, is wTO a more accurate measure for this? Figure 1 shows that while there is a very strong correlation between the pairwise correlation and the wTO, it is not absolute. Interestingly, for the lowest quality (10 measurements per gene), low-quality wTO outperforms the low-quality correlation as a predictor of the reference (high-quality) correlation, with a mean Spearman correlation of 0.465 between the pairwise low-quality wTO and reference bicor (vs 0.382 for low-quality bicor and reference bicor).

**Fig. 2:**
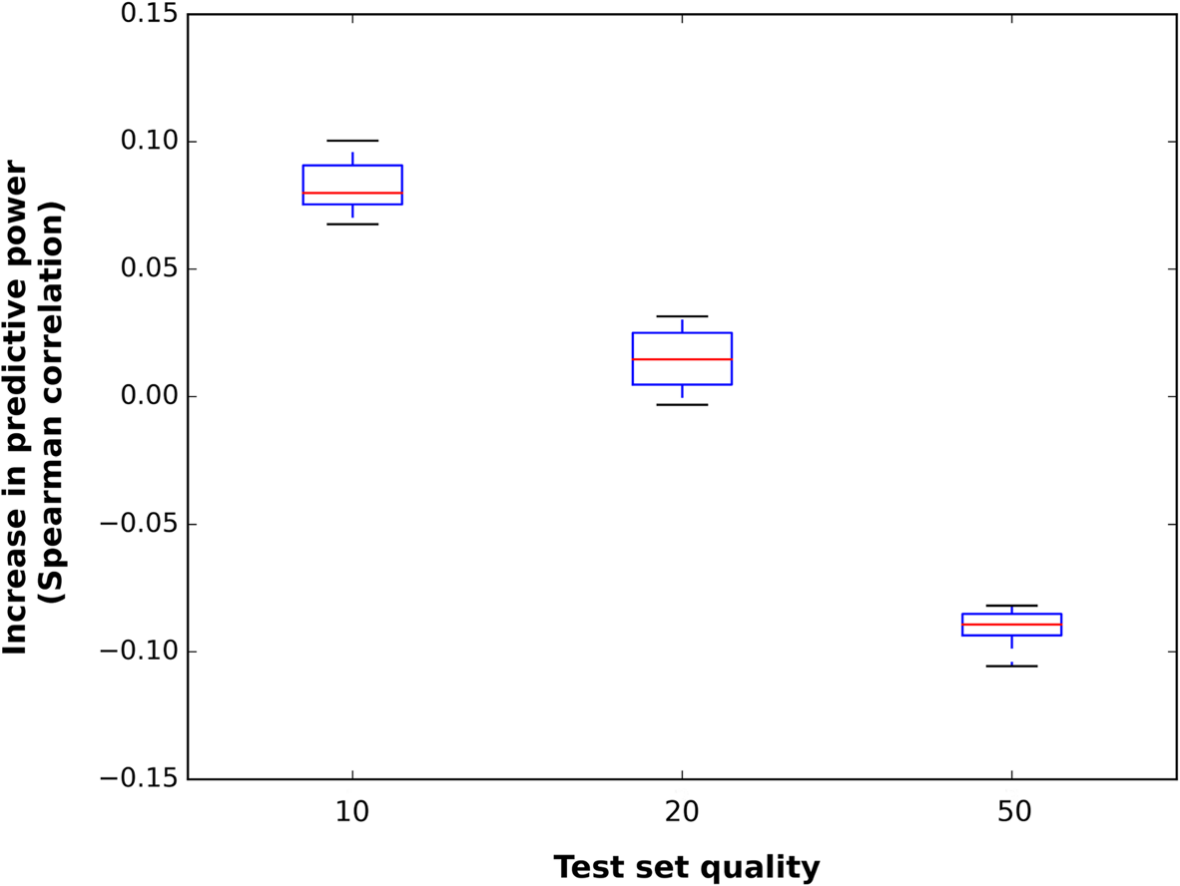
Net advantage of wTO over base bicor in terms of estimating reference bicor, as measured by difference in Spearman correlation. Boxes represent the spread of results for each of the 20 sets of 1000 genes at each given quality

This effect weakens with increasing data quality: while the mean performance is still marginally improved at the 20-point quality, (Spearman test: 0.532 for wTO versus 0.51 for bicor), the distributions overlap substantially, lying in the range of [0.508;0.547] for the wTO and [0.482;0.532] for the base bicor. However, we observe that within our ensemble of low-quality sets, the highest-performing sets tend to be the same for wTO and for base bicor. This is demonstrated in Fig. 2, where we plot the spread in net difference between each pair of Spearman tests for bicor vs. wTO over the three test set quality levels.

In other words, while we do see some base bicor sets outperform some wTO sets (at 20-point quality), wTO will generally outperform base bicor for the same set. Out of 20 sets, only three demonstrate a lower performance using wTO, and in all three cases the difference is marginal (0.003 being the largest difference in favor of base bicor on the Spearman test). As we increase the data quality to 50 points per gene, the positive effect of wTO has vanished entirely, and we see that the low-quality data set with only bicor correlation is in fact a better predictor of the reference correlation (Fig. 2).

As our wTO and base bicor values can be paired (since we compute both of them for each individual sample), we can apply the Wilcoxon signed-rank test to establish the level of statistical significance related to the improvements shown in Fig. 2. As shown in Table 1, we find a *p*-value of 4.0·10^−4^ in favor of wTO at 20-point quality. For the other two low-quality sets, the Wilcoxon test returns *p*=1.3·10^−4^, thus in favor of wTO at 10 points, and in favor of base bicor at 50 points. We note that the equal *p*-values at 10 and 50 points are a consequence of the nature of the Wilcoxon test as a non-parametric test, and that for these two qualities the same measure performs best in each of the 20 samples (wTO at 10 points, base bicor at 50).

**Table 1:** Wilcoxon test results for wTO versus base bicor performance using non-soft-thresholded networks, as shown in Fig. 2

Quality (points)	# of sets with superior wTO performance	*P*-value
10	20	1.3·10^−4^
20	17	4·10^−4^
50	0	1.3·10^−4^

The lower panel of Fig. 1 shows the Jaccard test for the top 1000 strongest edge pairs when comparing the test sets with the reference set. Somewhat surprisingly, in the test of low-quality correlation as predictor of reference correlation, the top-1000 strongest links when using a sub-sample set of size 50 shows a maximum Jaccard score of only 0.046. For the wTO-based networks, the Jaccard test accentuates the fidelity difference we have already observed, with a median score of 0.42.

**Fig. 3:**
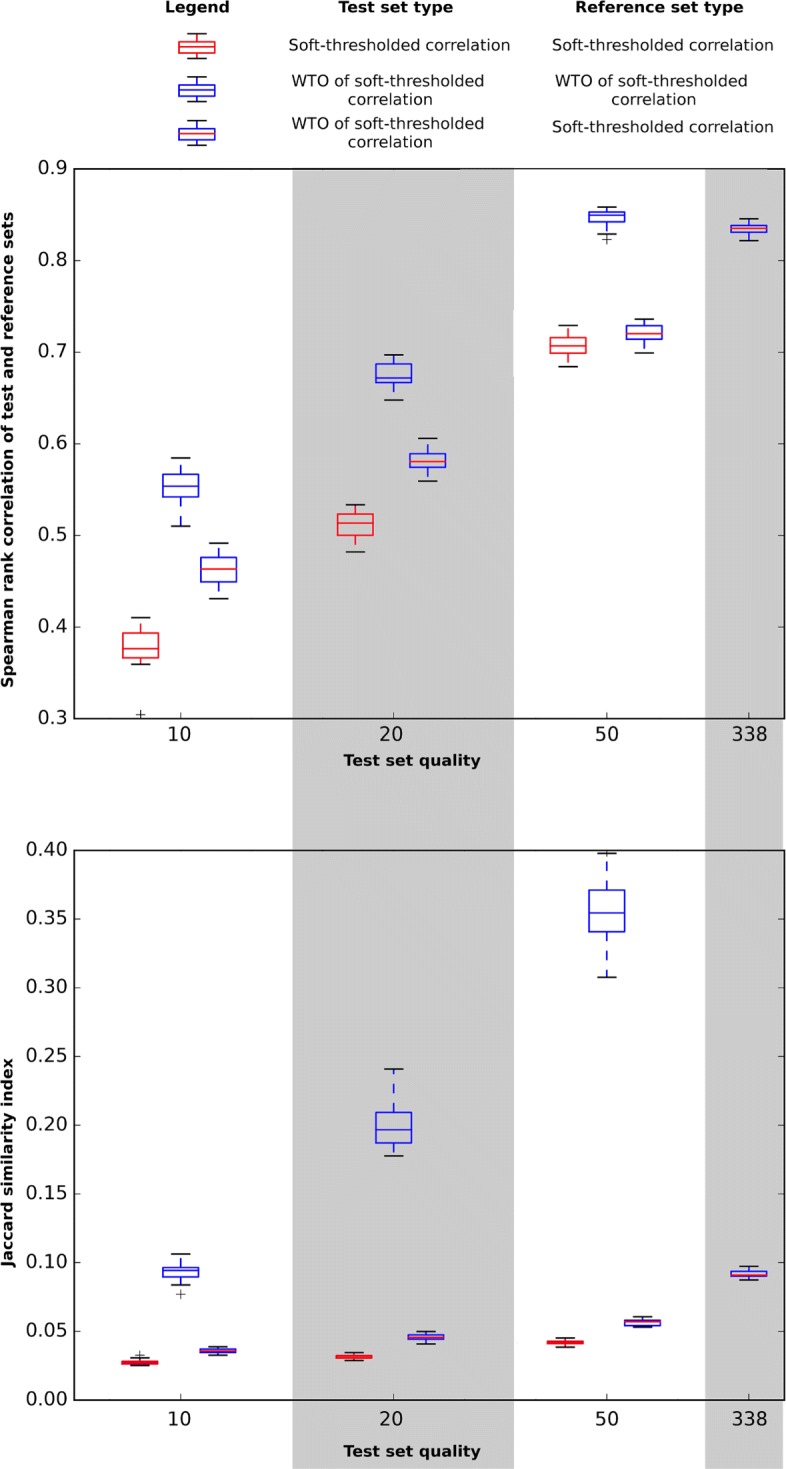
Comparison of fidelity to full-sample data between soft-thresholded correlation (bicor) and wTO of the soft-thresholded network. Note that soft-thresholding does not affect the relative ranking of base edge weights, and as such there is no difference between using a soft-thresholded base correlation and a non-soft-thresholded base correlation as the reference

Figure 3 shows, using both the Spearman test (upper panel) and the Jaccard test (lower panel), that applying a soft threshold to the bicor has some negative impact on the consistency of the wTO for all three low-quality sets. This is particularly evident for the 10- and 20 point measurement sets. Note that, since soft thresholding does not affect the relative ranking of network links (gene pairs), it does not affect the consistency of the non-wTO bicor network.

However, we see that soft-thresholding significantly increases consistency between wTO and base correlation for all low-quality sets. The improvement from soft thresholding is greater for higher-quality sets, partially off-setting the loss in predictive power seen in the non-soft-thresholded sets. Notably, soft-thresholded wTO consistently outperforms base bicor at 50-point quality, but not as much as it does for lower qualities. This is in contrast to the non-soft-thresholded case, where basic bicor outperforms wTO with regards to predicting reference bicor at 50-point quality. Similar to the non-soft-thresholded case, we find that despite the overlap for 50-point quality, the best-performing gene sets for base bicor are also the best-performing for soft-thresholded wTO, with soft-thresholded wTO outperforming base bicor for each of the 20 tested gene sets (as shown in Table 2, *p*=1.3·10^−4^, Wilcoxon test; see also Additional file 3: Performance improvement from WTO in a soft-thresholded network).

**Table 2:** Wilcoxon test results for wTO versus base bicor performance using soft-thresholded base correlation networks

Quality (points)	# of sets with superior wTO performance	*P*-value
10	20	1.3·10^−4^
20	20	1.3·10^−4^
50	0	1.3·10^−4^

As an additional test, we evaluated the performance of wTO by determining the expected number of false positives on gene expression sets consisting of identically distributed random data points. As we had previously determined that the choice of bicor or Spearman as the base correlation measure had a minimal impact on performance, we decided to use Spearman as the base correlation measure for this test. The major benefit of this choice was that since the Spearman correlation is a non-parametric measure, and only takes into account the relative ranking of genes, we could generate random data by any distribution we saw fit (requiring only that each data point was independent) without impacting the result. For the sake of simplicity, expression data was therefore drawn from a uniform distribution on [0,1]. By this process, we generated 20 groups of randomized data sets, containing 1000 genes (effectively, fixed numerical labels serving no other purpose than identification). In order to remain comparable to our analysis of whole-blood data, each of these 20 groups consist of four nested sets of 10, 20, 50 and 338 data points per gene.

In order to provide an equal basis for comparison of wTO and base correlation, we chose three pairs of cut-offs, corresponding to the 90th, 95th and 99th percentile of each of the Spearman correlation and wTO across our 20 reference (338-point) whole blood datasets. Any gene pair in the correlation and wTO networks produced from the randomized data sets (which, naturally, contains no true positives) was then flagged as a false positive if it exceeded the corresponding cut-offs.

As shown in Table 3, while base correlation returns a substantial number of false positives at lower qualities (predictably decreasing as the quality increases and the cut-off becomes more stringent), wTO performs far better, returning no false positives, even at the lowest cut-off chosen and lowest quality.

**Table 3:** False positive rates (FPR) for wTO and Spearman correlation networks obtained from random gene expression data

Percentile	Cut-off value (Spearman)	Cut-off value (wTO)	Quality (points)	FPR (Spearman)	FPR (wTO)
90	.5879	.4826	10	7.3·10^−2^	0
95	.6848	.5202	10	3.5·10^−2^	0
99	.8303	.6321	10	4.7·10^−3^	0
90	.5879	.4826	20	7.4·10^−3^	0
95	.6848	.5202	20	1.2·10^−3^	0
99	.8303	.6321	50	9.2·10^−7^	0
90	.5879	.4826	50	1.0·10^−5^	0
95	.6848	.5202	50	1.0·10^−7^	0
99	.8303	.6321	50	0	0
90	.5879	.4826	338	0	0
95	.6848	.5202	338	0	0
99	.8303	.6321	338	0	0

The “Percentile” column refers to the basis for false positive threshold, as the corresponding percentile of each metric in the whole-blood data set

### Resulting networks

In order to assess how the choice of wTO versus base correlation affects higher-order network characteristics rather than edge-wise characteristics, we calculated several properties of unweighted networks generated from the strongest 0.2*%* of gene pairs in each of our sets (using the Spearman correlation as the base correlation). We found that both the choice of score and the quality of the data have a strong effect on the number of nodes in the network (Additional files 4 and 5).

**Fig. 4:**
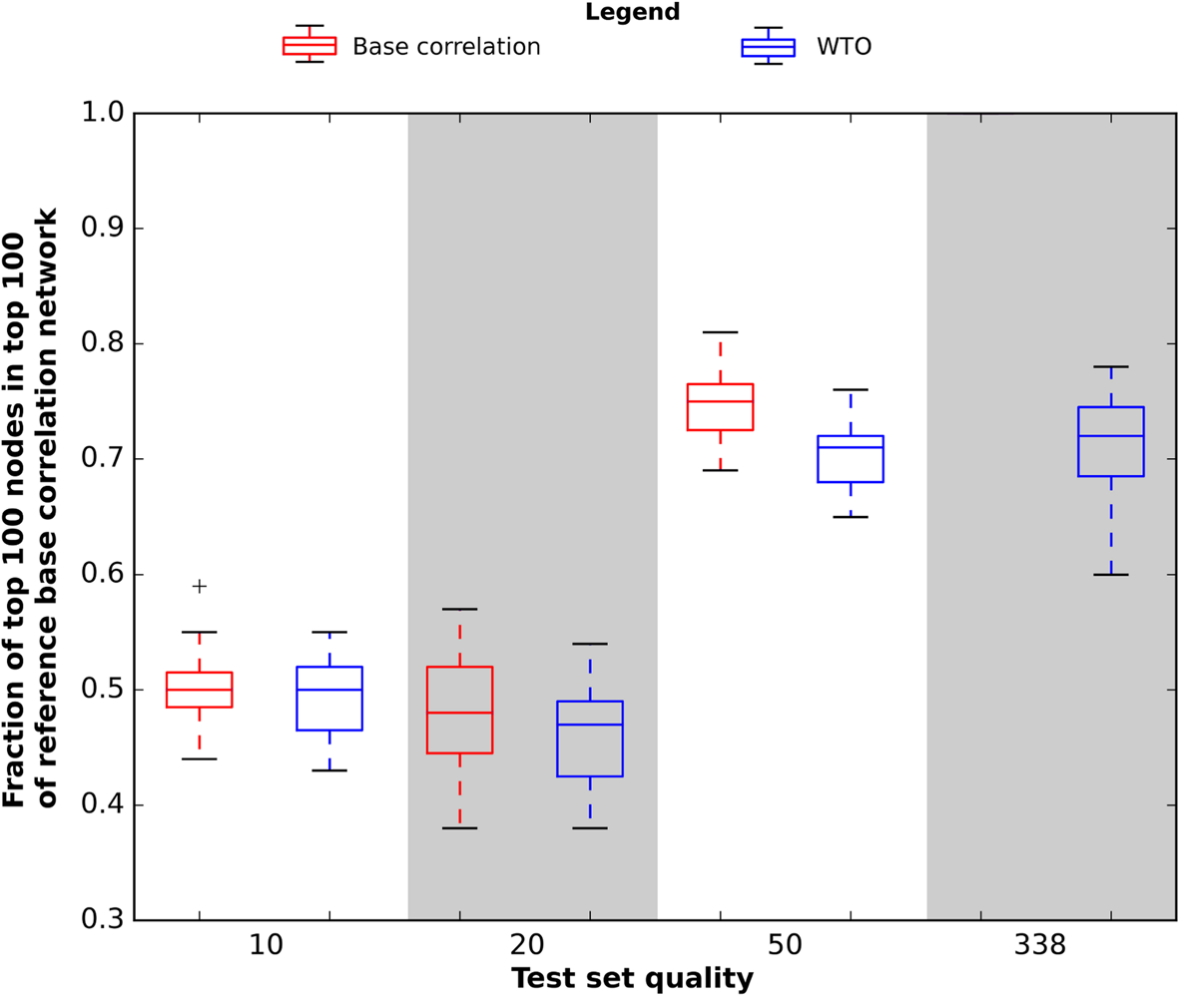
Similarity between low-quality hub nodes and hubs in the reference correlation network. The top 100 highest-connectivity (by node degree) nodes in each network were compared to the reference top 100 nodes in the full-quality network built from the base correlation matrix

As the number of edges is constant across all networks, these differences also correspond to differences in average degree of each node. Notably, we see that the choice of applying wTO as link weight reduces the number (and therefore increases the average connectivity) of nodes in the network. Interestingly, while the results are reasonably similar for the full-quality sets (median of 451 nodes for correlation, 515 for wTO), a reduction in quality has the opposite effect for wTO and base correlation: the former sees a reduction in the number of nodes, while the latter an increase. In order to evaluate how this might affect the top hubs present in each network, we identified the top 100 nodes by degree in each network and evaluated the proportion of these also present in the top 100 nodes of each of the reference networks. As shown in Fig. 4 and Additional file 6, we find that the choice between wTO and base bicor has minimal effect on the ability to replicate the top hubs by this approach.

Since community detection is one of the main motivations for WGCNA’s use of wTO, it is important to assess the community structure of the resulting networks. In fact, we find that using wTO has a dramatic impact on the community structure of the resulting network, with a substantial reduction of the number of modules identified across all set qualities. Furthermore, the wTO-based networks contain fewer distinct modules (and this number remains low and constant across our tested quality levels) than the when using the base correlation, for which the number of identified modules increases as the data quality is reduced (Additional files 7 and 8). We also note that the modularity of the best partition, as identified by the Louvain algorithm [26], is lower for high-quality base correlation sets, and for wTO-based networks as a whole (Additional files 9 and 10). A possible explanation for this is that wTO tends to filter smaller isolated gene groups (whether the result of noise or not) in favor of densely interconnected cores, in which boundaries between communities are less clear.

We also note substantial differences in local topologies, with the wTO-based networks predictably exhibiting substantially higher clustering (tendency for nodes to share neighbors) than the base correlation networks (Additional files 11 and 12). Finally, we find a marked difference between correlation and wTO-based networks with respect to degree assortativity: the correlation-based networks exhibit positive assortativity (well-connected nodes connect to well-connected nodes, indicating a rich-club structure), in particular for the lower-quality sets, while the wTO-based networks exhibit a more consistent negative assortativity across the board, indicating a hub-and-spoke structure in the network (Additional files 13 and 14).

## Discussion

Our study confirms that by including information from the local neighborhood of a pair of correlated genes, wTO has the potential for being a substantial improvement on pairwise correlations as a measure of co-expression, in particular for data sets with very few measurements per gene. While this is in line with common practice, it is not a trivial finding: certainly, wTO is often found to be strongly correlated to edge weights, such as social networks [30]. The latter is generally a consequence of transitivity of edges: for instance, if person A is friends with person B and with person C, B and C are usually more likely to be friends.

In many cases, such as the aforementioned social networks, this edge-transitivity is a reflection of the dynamics forming the system. Examples of such could be similar interests and geographical proximity, themselves being transitive attributes). However the expectation of transitivity is not an absolute in these systems, as it is certainly conceivable for A to be very good friends with both B and C, without B or C having ever met each other. Similarly, we could certainly expect similar topological cases in co-expression networks, consisting of sets of three or more genes involved in similar processes, which would rely on the expression of all these genes to function and, thus, forming cliques with the result being noticeable topological overlap.

For correlations, however, the transitivity does not just reflect the dynamics behind the data, but it is also mathematically necessary by nature. Consider the case of gene A being strongly correlated with both of genes B and C. In this situation, genes B and C must necessarily be strongly correlated as well. What this means is that, even for links which are coincidentally correlated, one would expect a large topological overlap, as coincidental correlation with another node necessarily implies some degree of (similarly coincidental) correlation with its neighbors. Consequently, we would expect that spuriously correlated gene pairs could also show high topological overlap, and as a direct effect, that the topological overlap measure should be just as vulnerable to noise resulting from low sample sizes. Because of this, it might not immediately follow that the benefits of using topological overlap, as seen in a wide variety of network analyses, also apply to correlation networks.

However, we find that topological overlap network is much more robust than a base correlation network. Additionally, for the case of low-quality data sets, wTO is a substantially better predictor of reference correlation than the correlation itself (despite the fact that it actually estimates a separate variable). The effect weakens with increasing quality. For very high-quality sets, it appears that wTO becomes a worse estimator of the “infinite-set” correlation. Since the wTO measure is a non-linear transformation of a correlation link-weight by drawing upon the joint neighborhood two genes, this is as expected.

In terms of network structure, our results show a complex reality where it is necessary to evaluate multiple effects against each other. Notably, we find that many of the key network characteristics show substantial change when building networks from wTO scores rather than base correlations, and hence, that choosing one over the other may have a notable impact on certain types of results. This effect is most marked at low qualities (few data samples), as many system-level scores in correlation-based networks (assortativity, clustering, modularity) exhibit values more similar to the wTO networks as the quality increases. However, these metrics (most notably the assortativity) still show clear differences between both types of networks at full quality, and there is no evidence that further increase in quality would result in convergence to a single value.

## Conclusions

In this paper, we have investigated the ability of the weighted topological overlap method to generate gene co-expression networks with improved fidelity in situations of low measurement numbers. We find that, with respect to edge interactions, the wTO method is systematically able to improve upon low-quality data, even to provide a better prediction of reference correlations for sample sets consisting of only 10 or 20 measurements per gene. These results reinforce the credibility of wTO as a valuable metric when evaluating gene co-expression networks, as is customary by established software packages such as WGCNA. However, we also find that using wTO as a replacement for correlation with respect to building networks when employing a cut-off value can have substantial effects on the topology of the resulting networks, particularly with respect to modularity.

Interestingly, we find that soft-thresholding reduces the robustness of wTO for low-quality sets, despite otherwise increasing its predictive power with regards to base correlation. While this demonstrates the potential usefulness of the well established combined wTO/soft-thresholding approach, it also highlights a potential weakness, which might be of concern for certain applications.

## Additional files

Additional file 1 Performance test of wTO against base Spearman for mouse data. Comparison of fidelity to full-sample data between non-modified pairwise correlations (Spearman) and wTO of the bicor network, according to two tests: Spearman rank correlation of edge pairs and Jaccard similarity of the top 1000 edge pairs. Similar to Fig. 1, using Spearman instead of bicor as the base edge weight. Similar to Additional file 1 but computed from the GSE26500 data set from murine brains. (PNG 226 kb)

Additional file 2 Performance test of wTO against base Spearman for human data. Comparison of fidelity to full-sample data between non-modified pairwise correlations (Spearman) and wTO of the bicor network, according to two tests: Spearman rank correlation of edge pairs and Jaccard similarity of the top 1000 edge pairs. Similar to Fig. 1, using Spearman instead of bicor as the base edge weight. (PNG 171 kb)

Additional file 3 Performance improvement from WTO in a soft-thresholded network. Net advantage of wTO over base bicor in terms of estimating reference bicor, as measured by difference in Spearman correlation. Boxes represent the spread of results for each of the 20 sets of 1000 genes at each given quality. Similar to Fig. 2, using soft-thresholded bicor instead of base bicor as the base edge weight. (PNG 107 kb)

Additional file 4 Number of nodes in networks obtained from human whole blood. (PNG 107 kb)

Additional file 5 Number of nodes in networks obtained from murine brains. (PNG 87 kb)

Additional file 6 Fraction of top 100 nodes (by degree) in top 100 of nodes of the reference correlation network. (PNG 118 kb)

Additional file 7 Number of distinct communities in the optimal partition identified by the Louvain community detection algorithm in networks obtained from human whole blood. (PNG 86 kb)

Additional file 8 Number of distinct communities in the optimal partition identified by the Louvain community detection algorithm in networks obtained from murine brains. (PNG 88 kb)

Additional file 9 Modularity score of the optimal partition identified by the Louvain community detection algorithm in networks obtained from human whole blood. (PNG 106 kb)

Additional file 10 Modularity score of the optimal partition identified by the Louvain community detection algorithm in networks obtained from murine brains. (PNG 87 kb)

Additional file 11 Average clustering coefficients in networks obtained from human whole blood. (PNG 101 kb)

Additional file 12 Average clustering coefficients in networks obtained from murine brains. (PNG 102 kb)

Additional file 13 Degree assortativity coefficients for networks obtained from human brains. (PNG 106 kb)

Additional file 14 Degree assortativity coefficients for networks obtained from murine brains. (PNG 107 kb)

## Abbreviations

bicor: Biweight midcorrelation
WGCNA: Weighted gene co-expression network analysis, an R package for gene co-expression analysis
wTO: weighted Topological Overlap, defined in Eq. 1
