# Supplementary figures and images for: Assessment of weighted topological overlap (wTO) to improve fidelity of gene co-expression networks
Source: BMC Bioinformatics. 2019 Jan 28;20:58. doi: 10.1186/s12859-019-2596-9 (PMC6350380; doi:10.1186/s12859-019-2596-9)

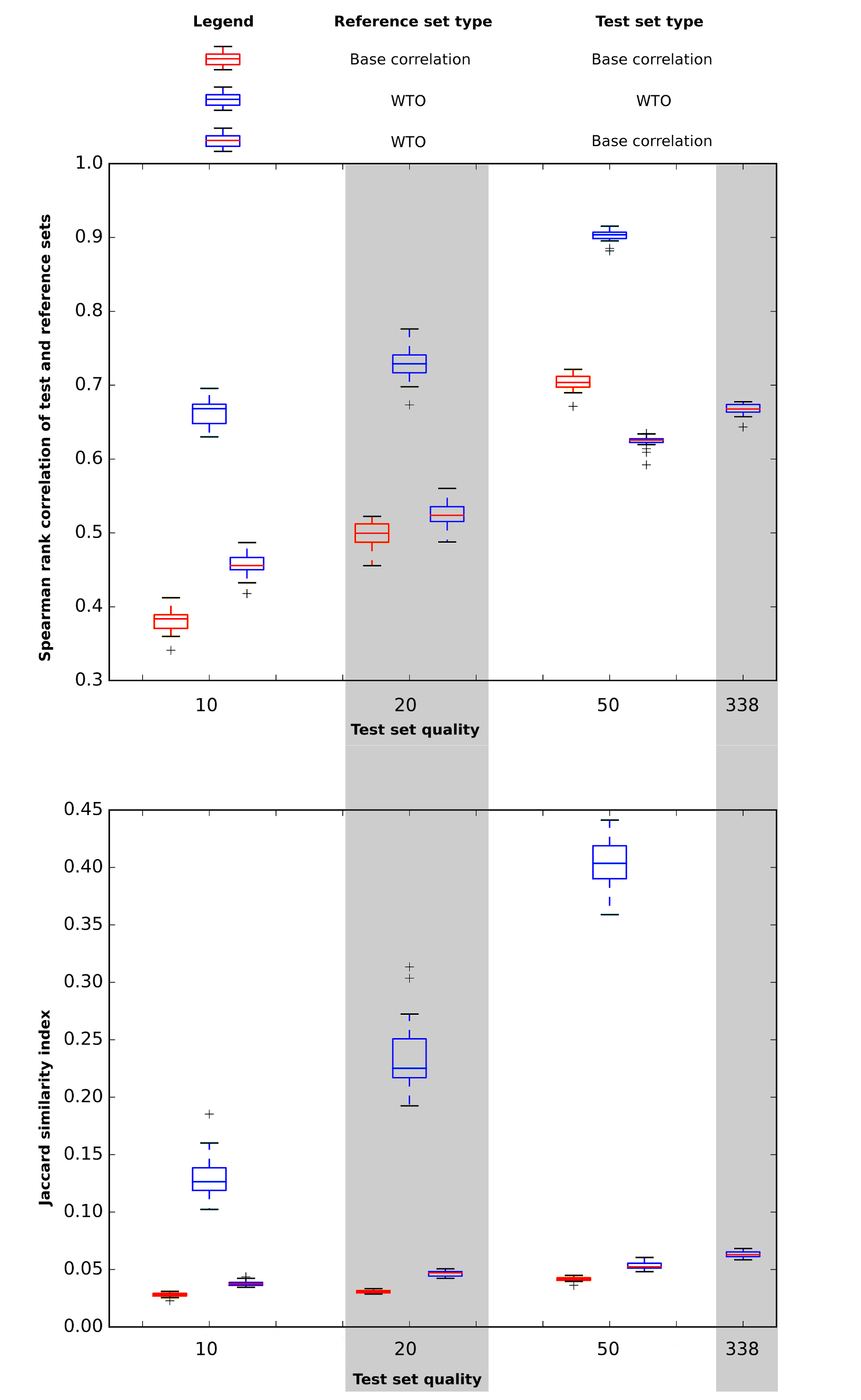

Supplement: Supplementary file 1 — Performance test of wTO against base Spearman for mouse data. Comparison of fidelity to full-sample data between non-modified pairwise correlations (Spearman) and wTO of the bicor network, according to two tests: Spearman rank correlation of edge pairs and Jaccard similarity of the top 1000 edge pairs. Similar to Fig. 1, using Spearman instead of bicor as the base edge weight. Similar to Additional file 1 but computed from the GSE26500 data set from murine brains. (PNG 226 kb) [file 12859_2019_2596_MOESM1_ESM.png]

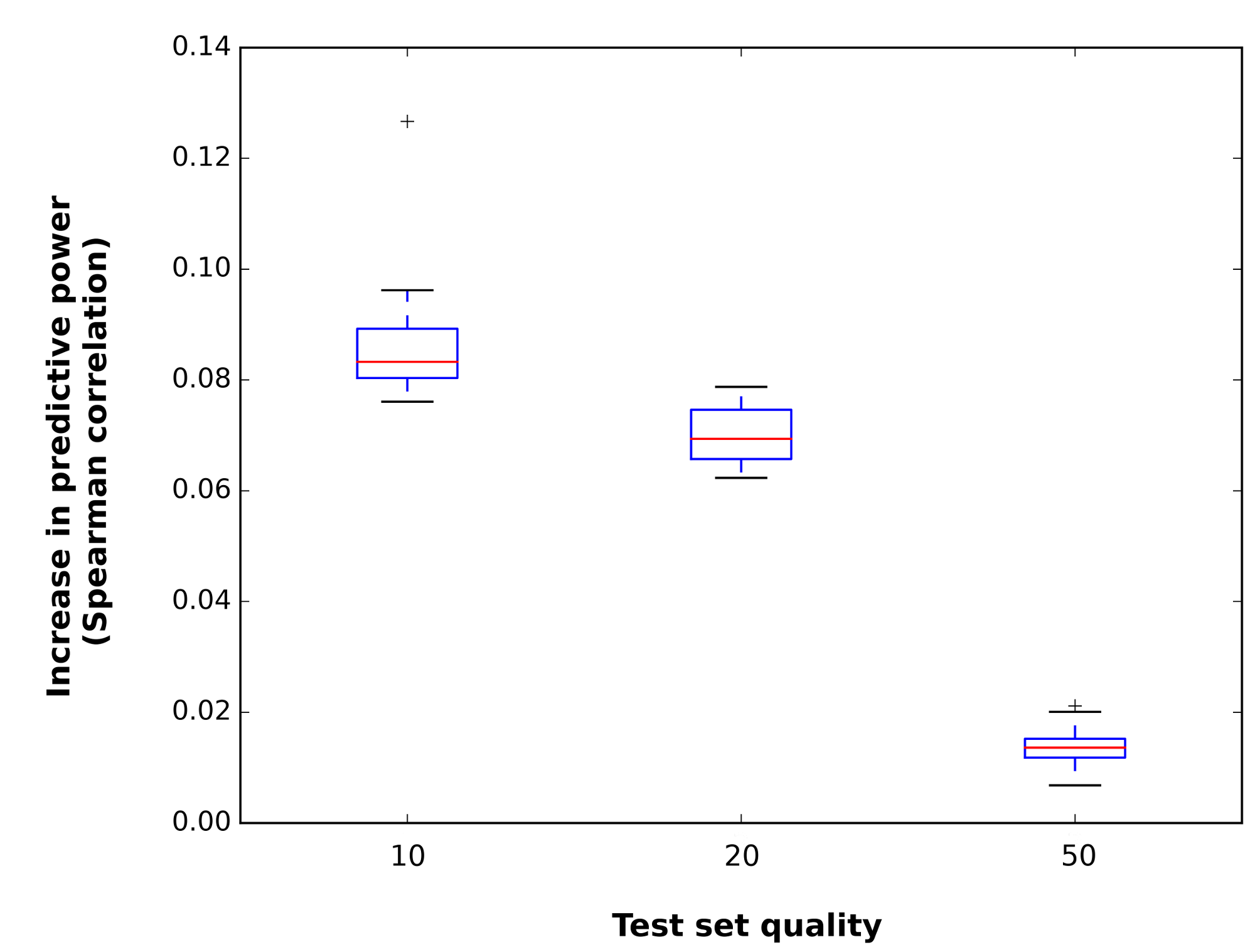

Supplement: Supplementary file 2 — Performance test of wTO against base Spearman for human data. Comparison of fidelity to full-sample data between non-modified pairwise correlations (Spearman) and wTO of the bicor network, according to two tests: Spearman rank correlation of edge pairs and Jaccard similarity of the top 1000 edge pairs. Similar to Fig. 1, using Spearman instead of bicor as the base edge weight. (PNG 171 kb) [file 12859_2019_2596_MOESM2_ESM.png]

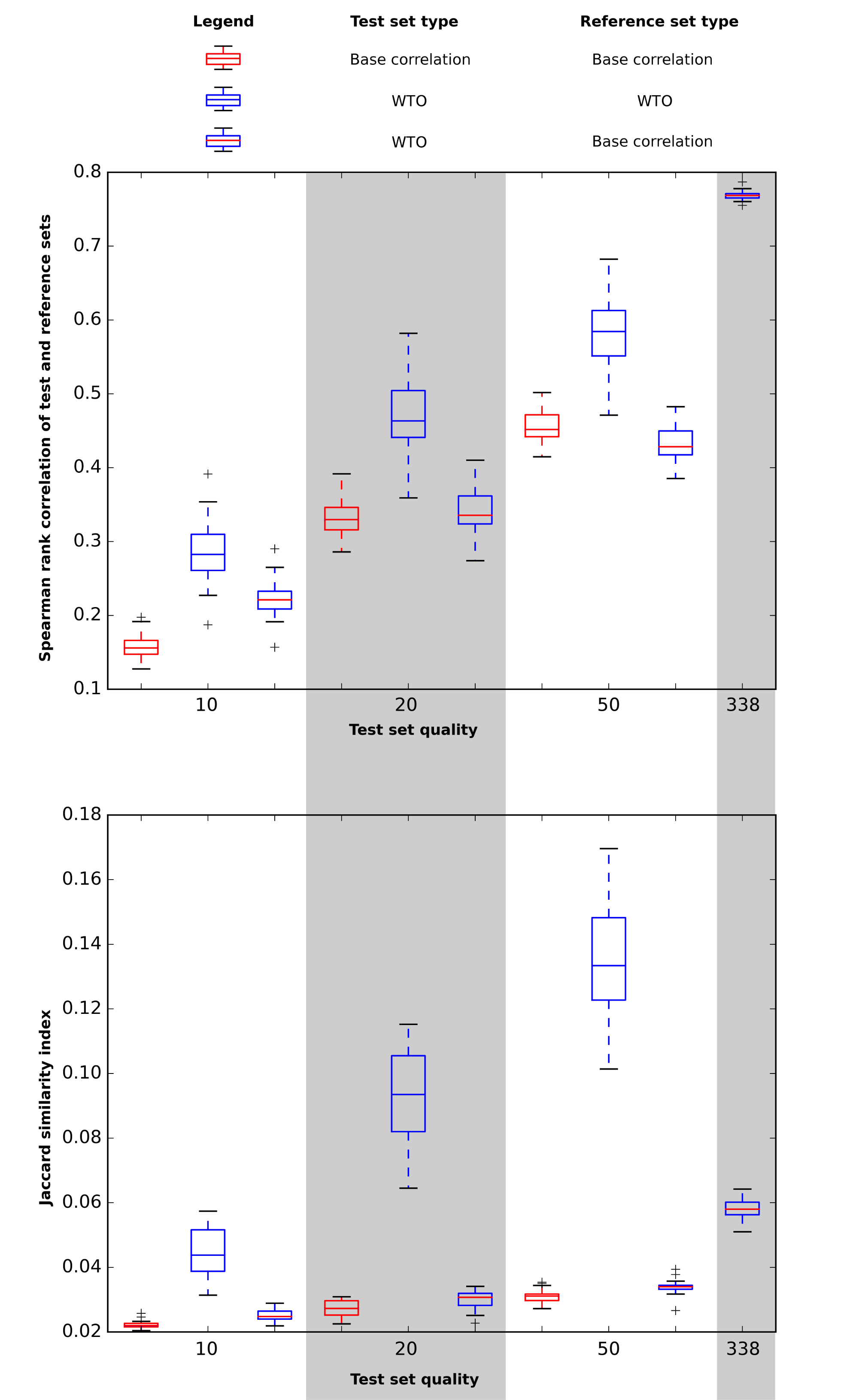

Supplement: Supplementary file 3 — Performance improvement from WTO in a soft-thresholded network. Net advantage of wTO over base bicor in terms of estimating reference bicor, as measured by difference in Spearman correlation. Boxes represent the spread of results for each of the 20 sets of 1000 genes at each given quality. Similar to Fig. 2, using soft-thresholded bicor instead of base bicor as the base edge weight. (PNG 107 kb) [file 12859_2019_2596_MOESM3_ESM.png]

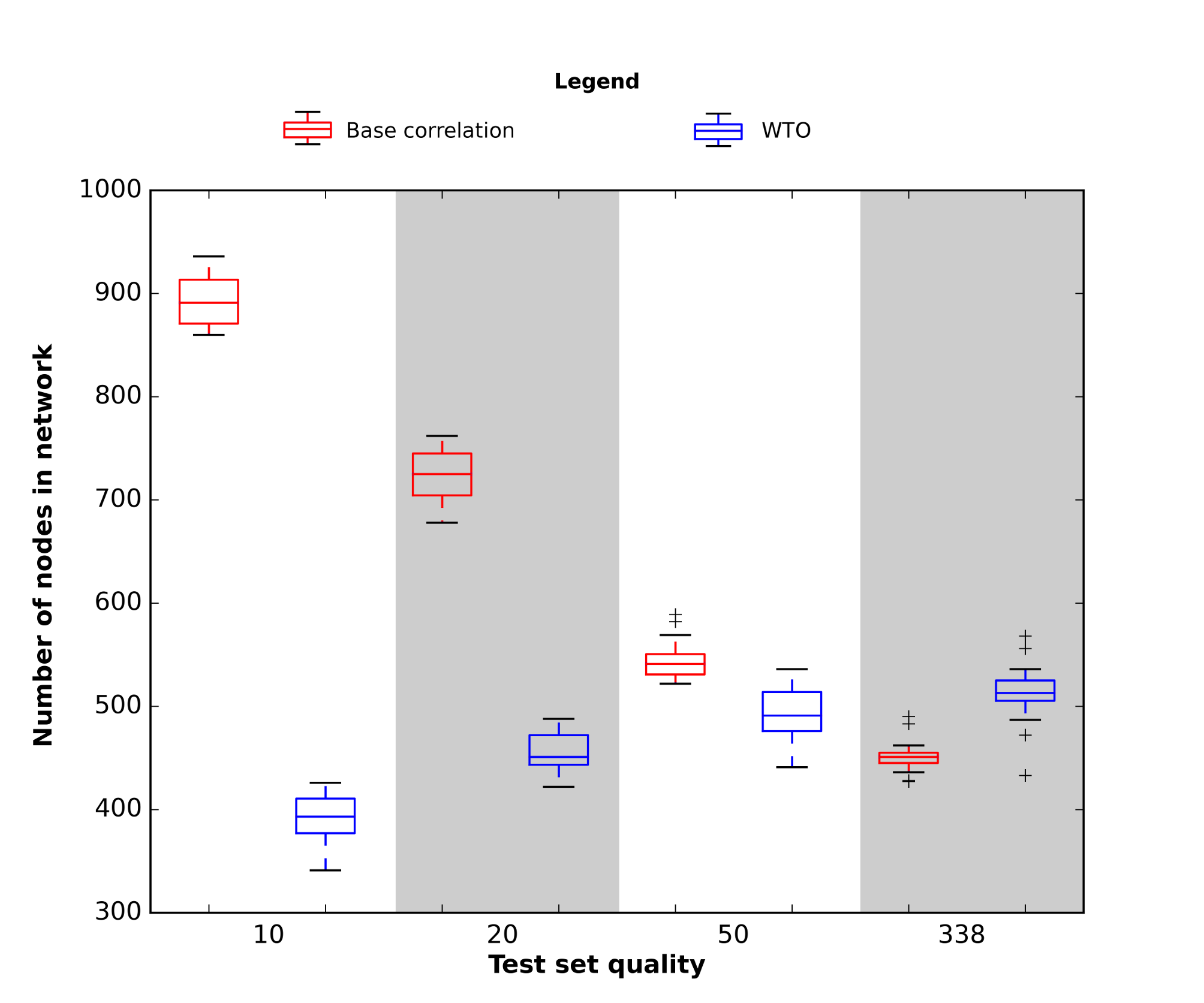

Supplement: Supplementary file 4 — Number of nodes in networks obtained from human whole blood. (PNG 107 kb) [file 12859_2019_2596_MOESM4_ESM.png]

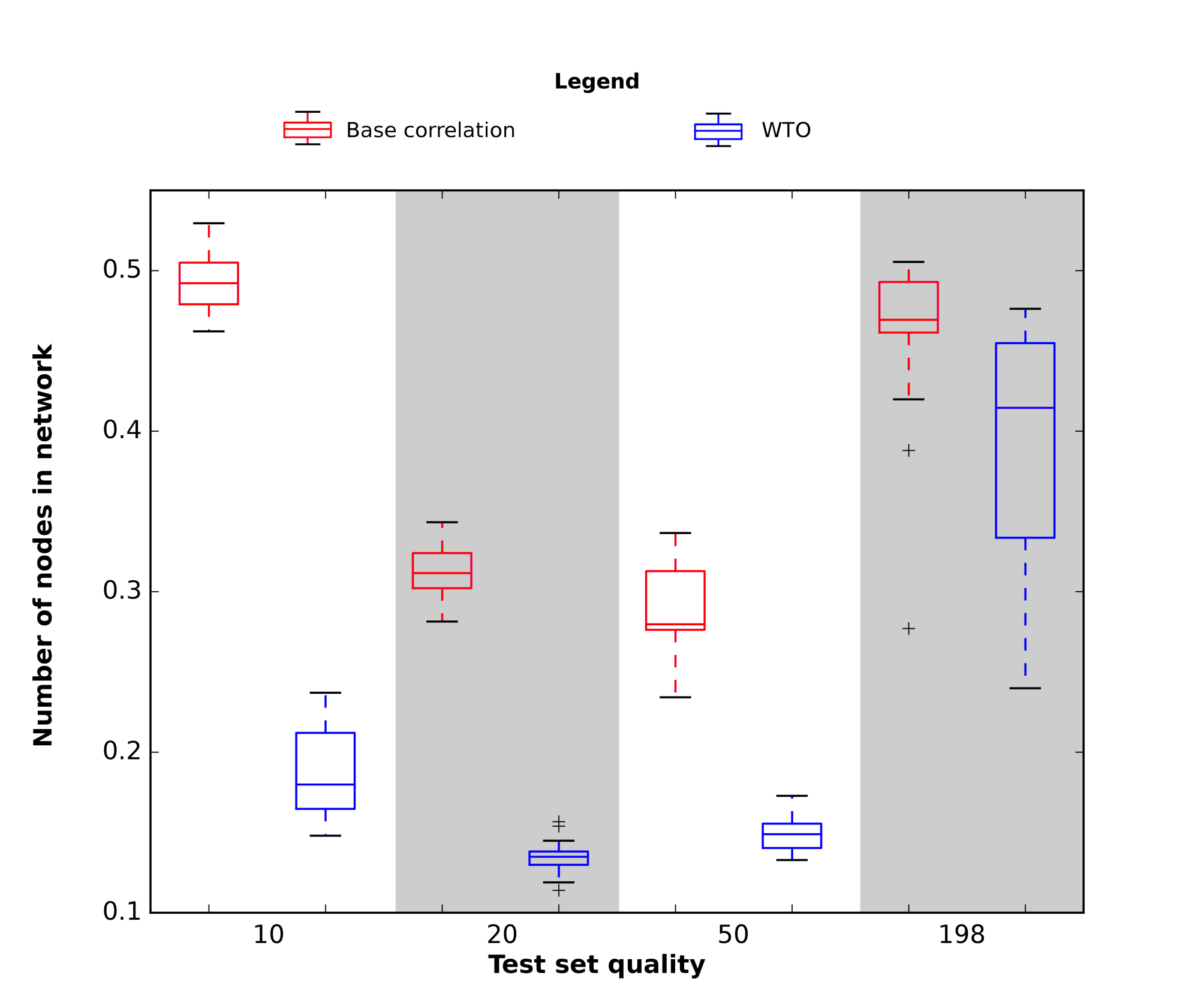

Supplement: Supplementary file 5 — Number of nodes in networks obtained from murine brains. (PNG 87 kb) [file 12859_2019_2596_MOESM5_ESM.png]

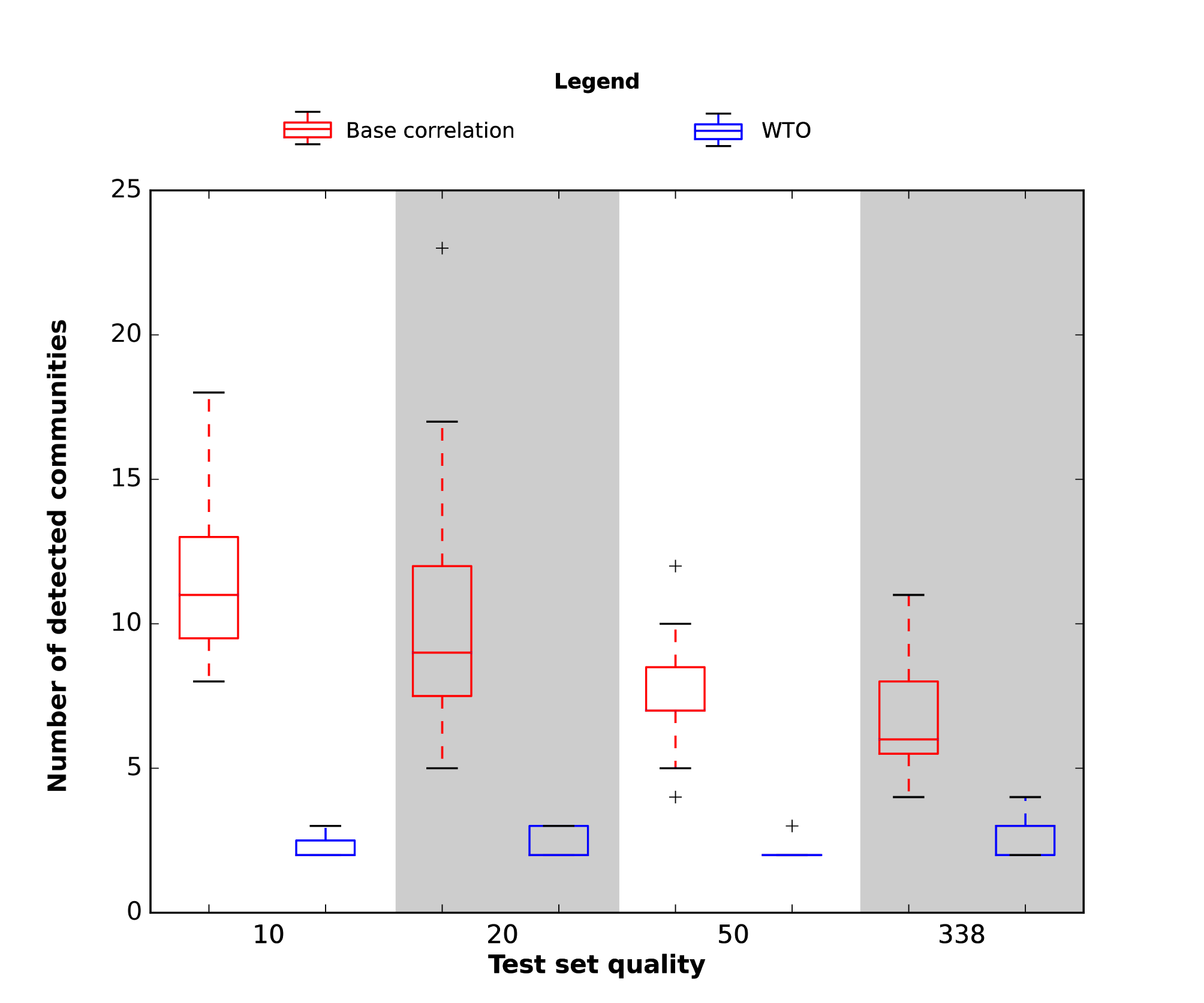

Supplement: Supplementary file 6 — Fraction of top 100 nodes (by degree) in top 100 of nodes of the reference correlation network. (PNG 118 kb) [file 12859_2019_2596_MOESM6_ESM.png]

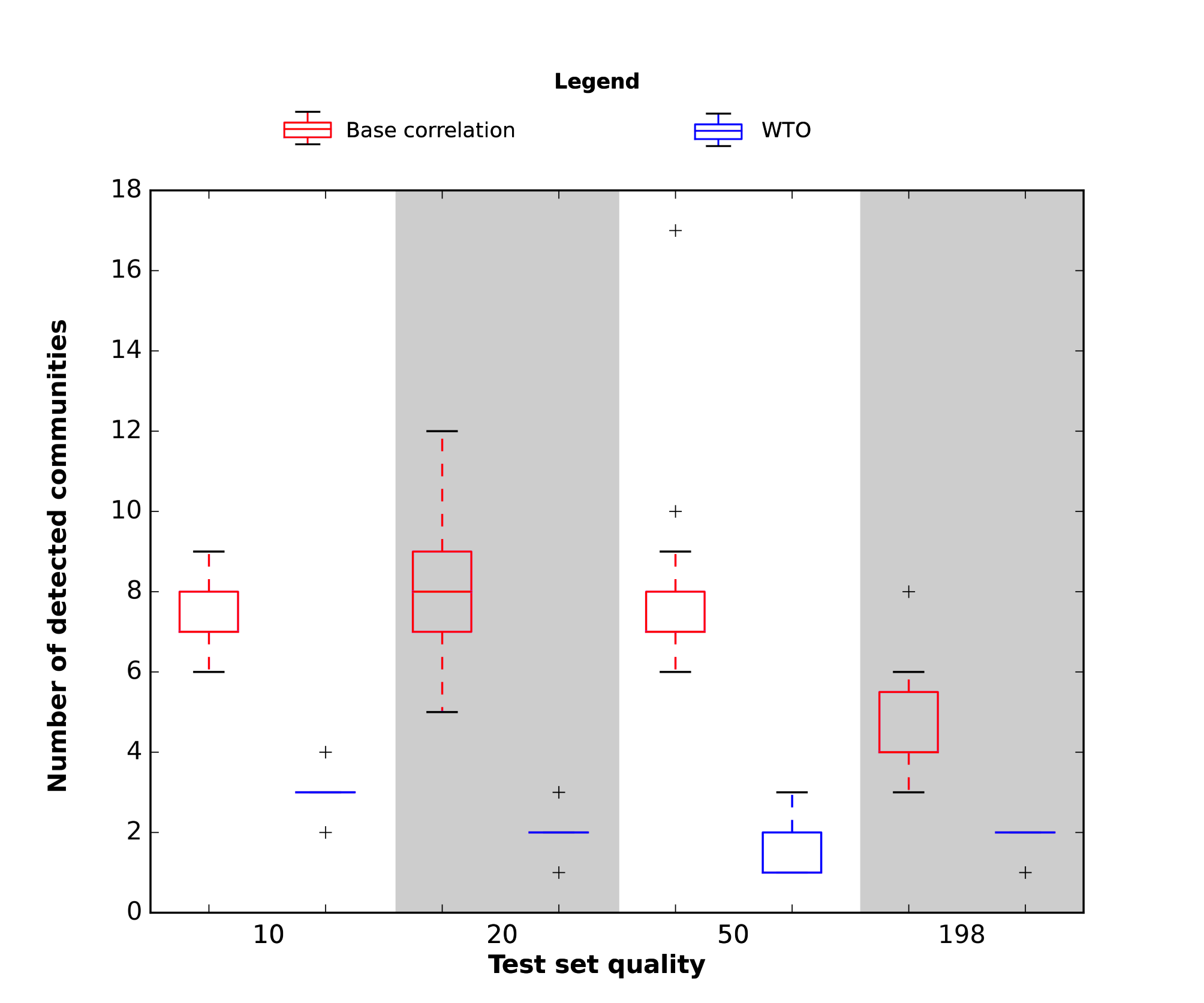

Supplement: Supplementary file 7 — Number of distinct communities in the optimal partition identified by the Louvain community detection algorithm in networks obtained from human whole blood. (PNG 86 kb) [file 12859_2019_2596_MOESM7_ESM.png]

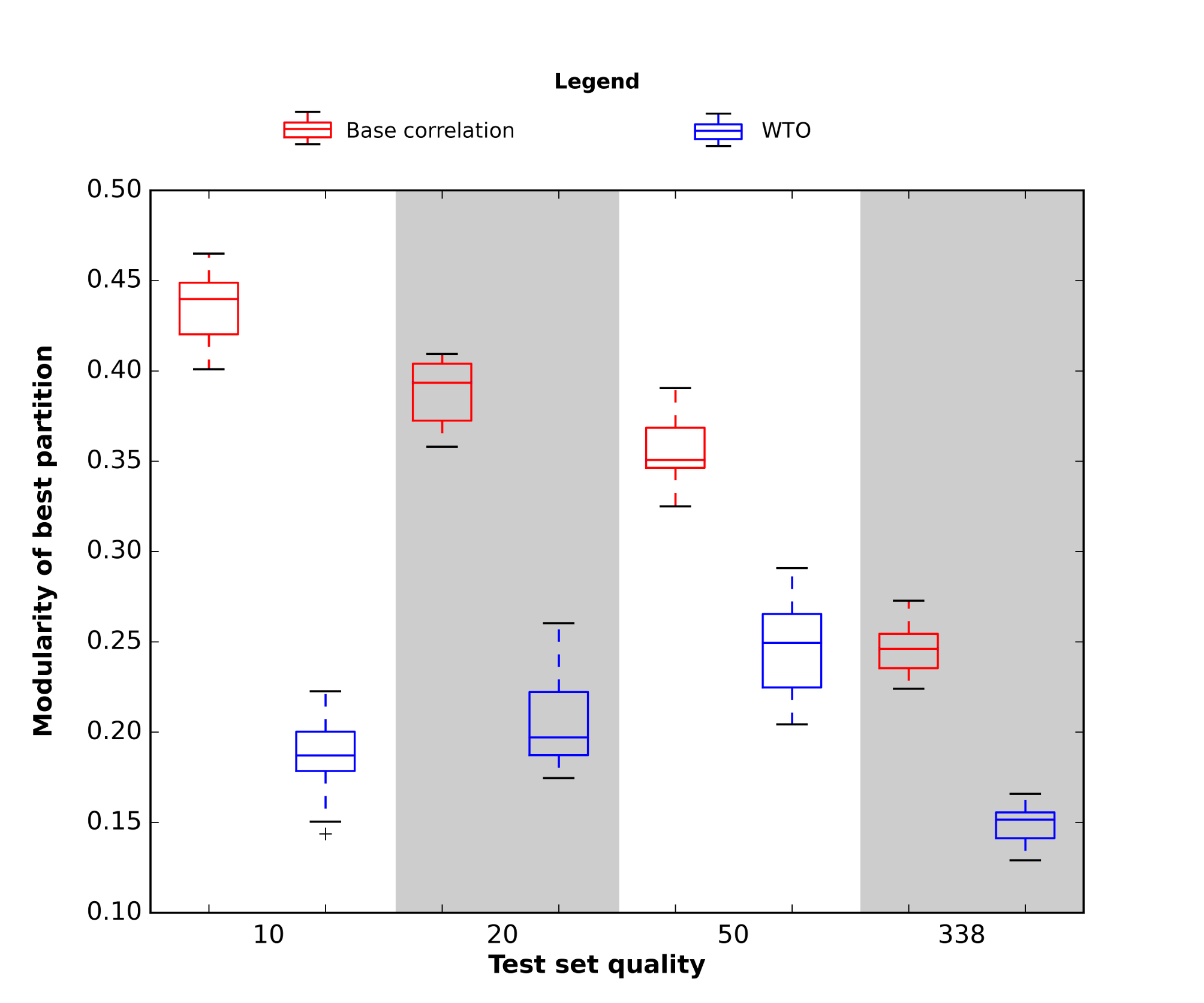

Supplement: Supplementary file 8 — Number of distinct communities in the optimal partition identified by the Louvain community detection algorithm in networks obtained from murine brains. (PNG 88 kb) [file 12859_2019_2596_MOESM8_ESM.png]

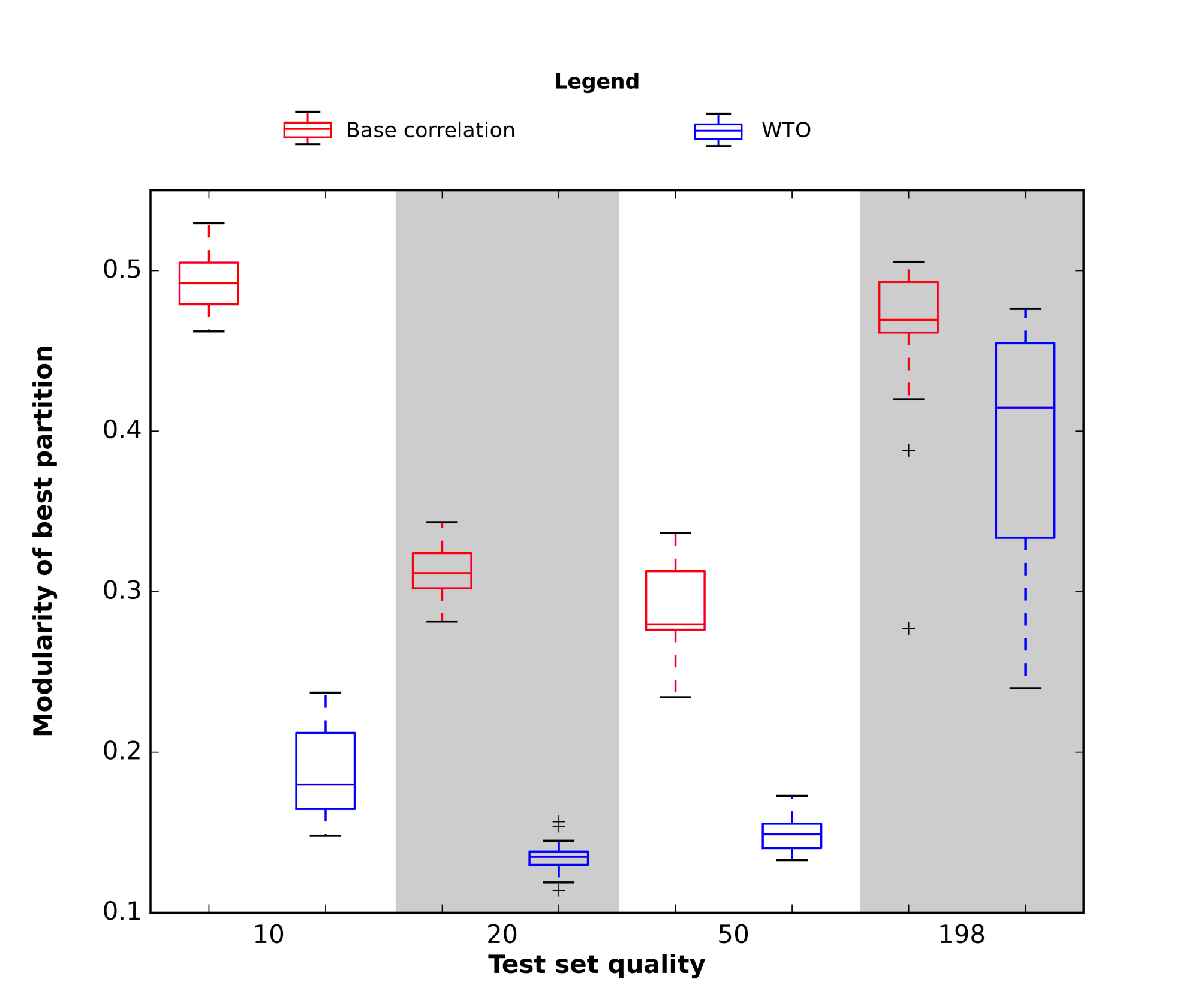

Supplement: Supplementary file 9 — Modularity score of the optimal partition identified by the Louvain community detection algorithm in networks obtained from human whole blood. (PNG 106 kb) [file 12859_2019_2596_MOESM9_ESM.png]

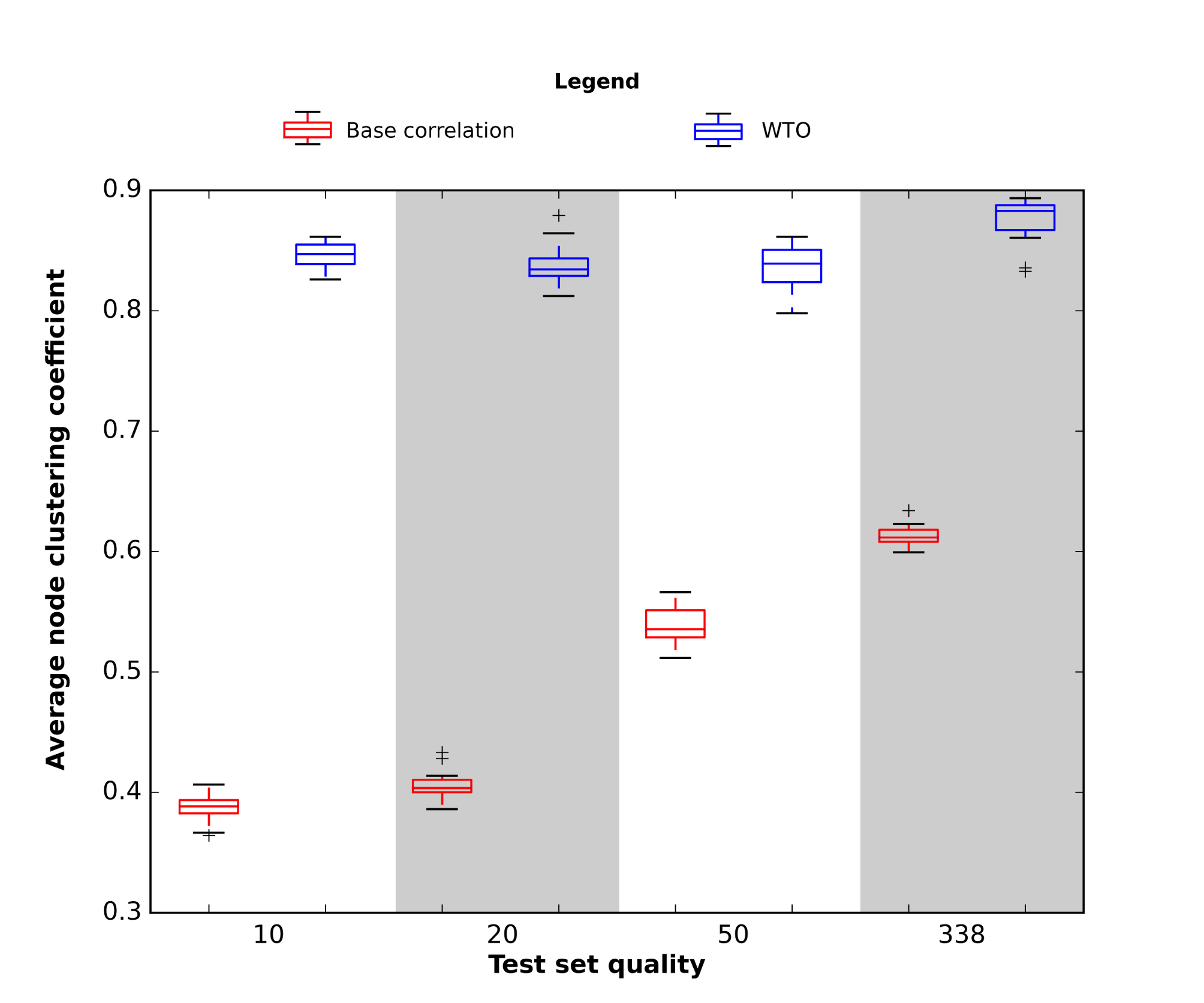

Supplement: Supplementary file 10 — Modularity score of the optimal partition identified by the Louvain community detection algorithm in networks obtained from murine brains. (PNG 87 kb) [file 12859_2019_2596_MOESM10_ESM.png]

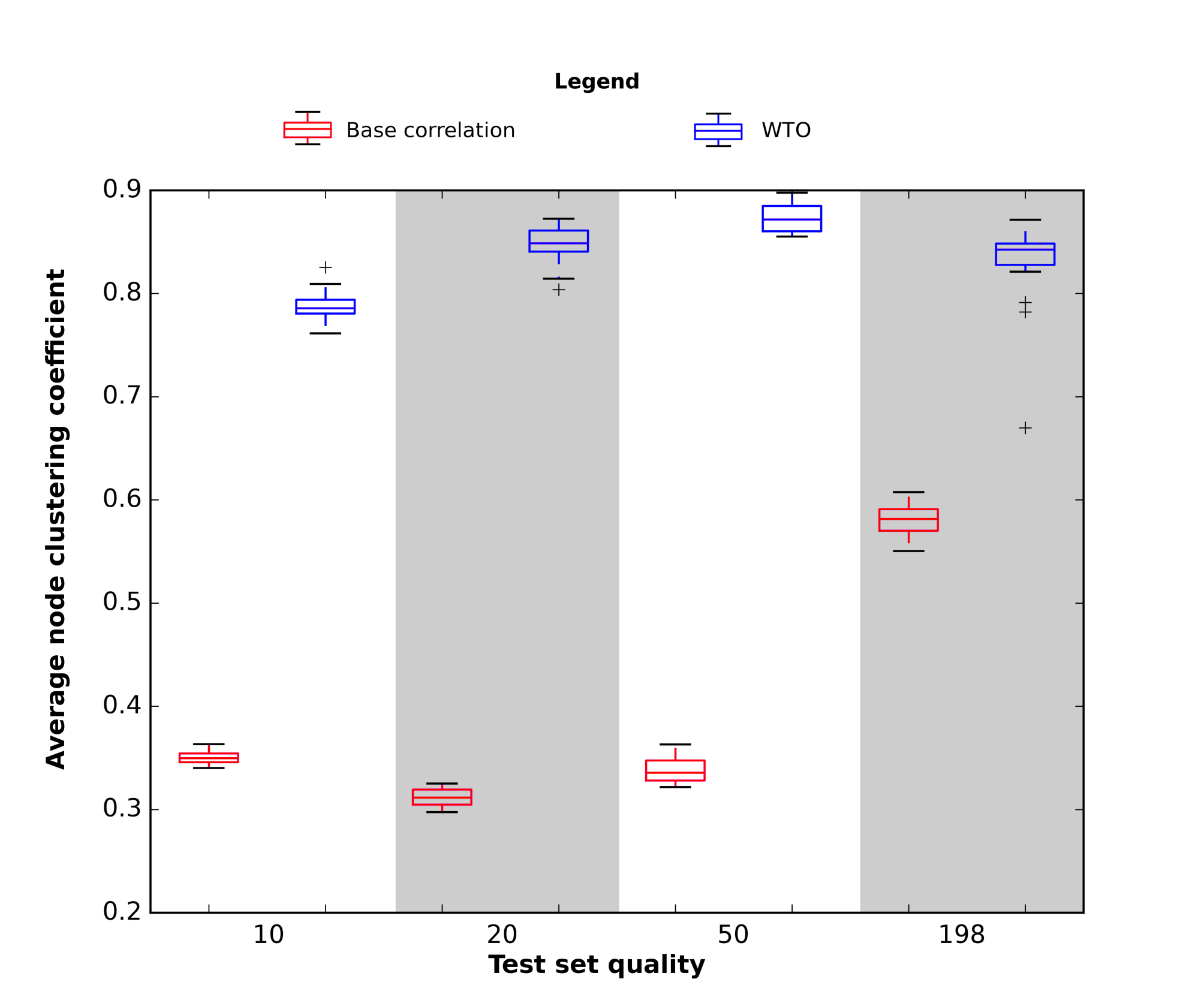

Supplement: Supplementary file 11 — Average clustering coefficients in networks obtained from human whole blood. (PNG 101 kb) [file 12859_2019_2596_MOESM11_ESM.png]

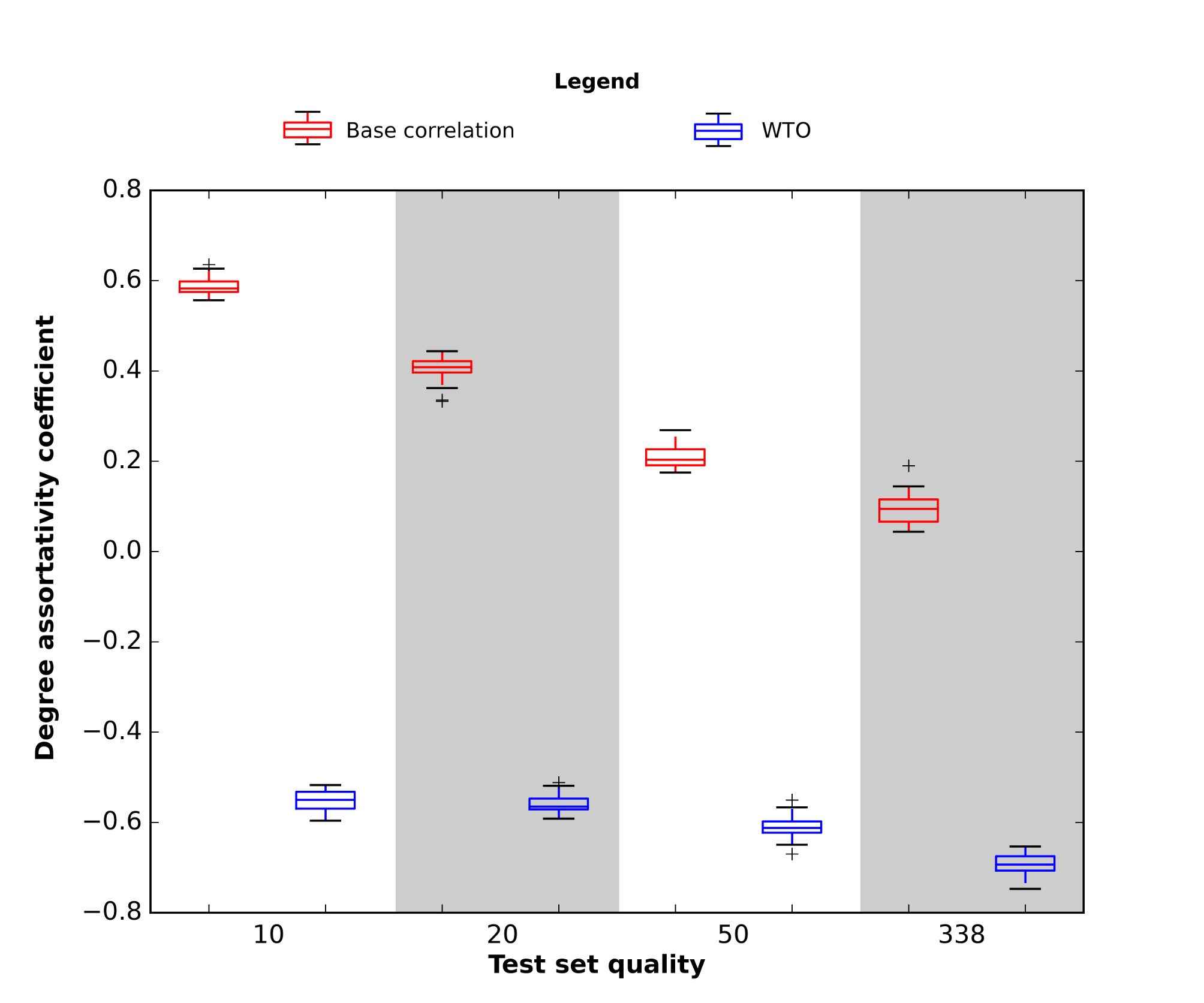

Supplement: Supplementary file 12 — Average clustering coefficients in networks obtained from murine brains. (PNG 102 kb) [file 12859_2019_2596_MOESM12_ESM.png]

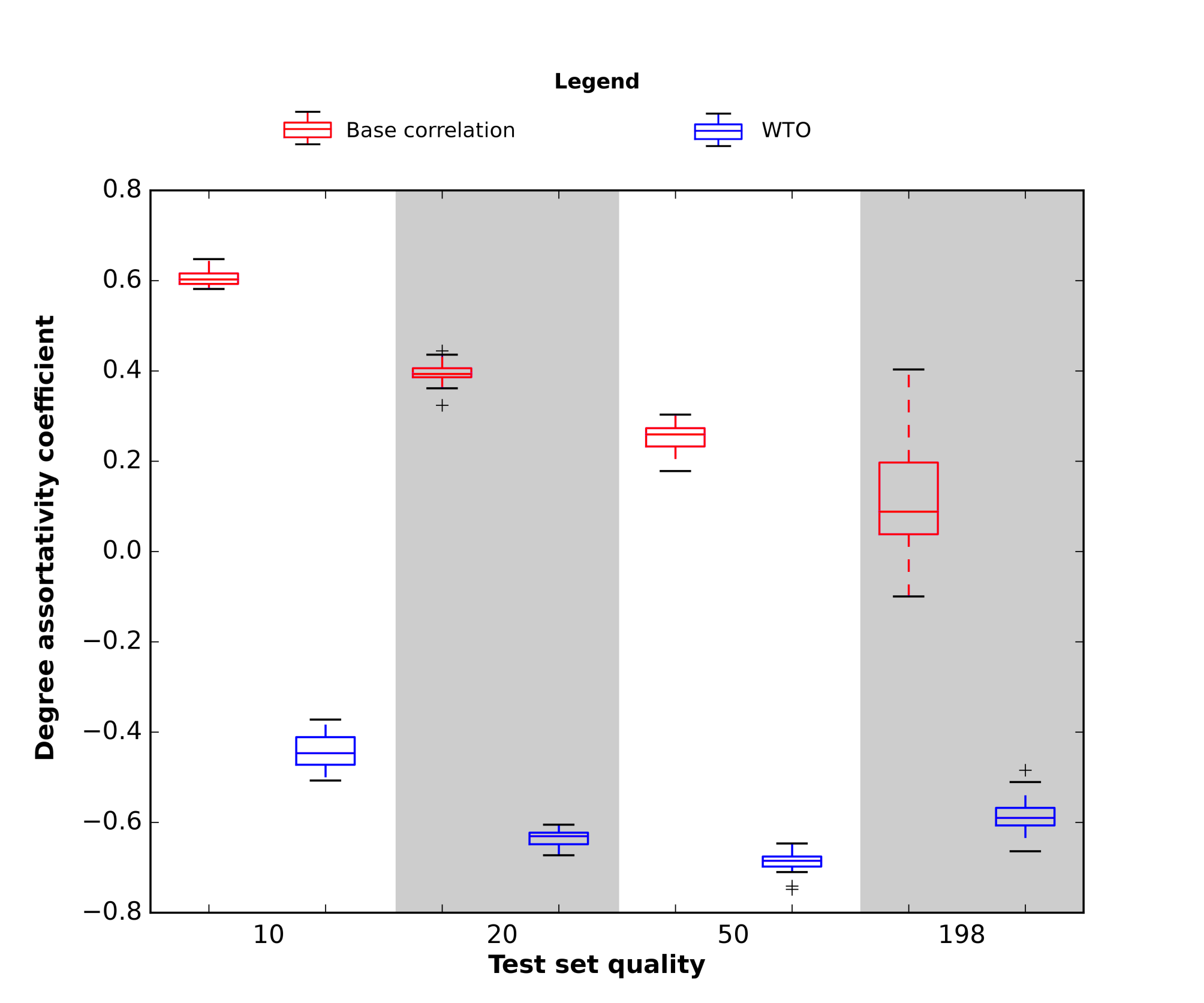

Supplement: Supplementary file 13 — Degree assortativity coefficients for networks obtained from human brains. (PNG 106 kb) [file 12859_2019_2596_MOESM13_ESM.png]

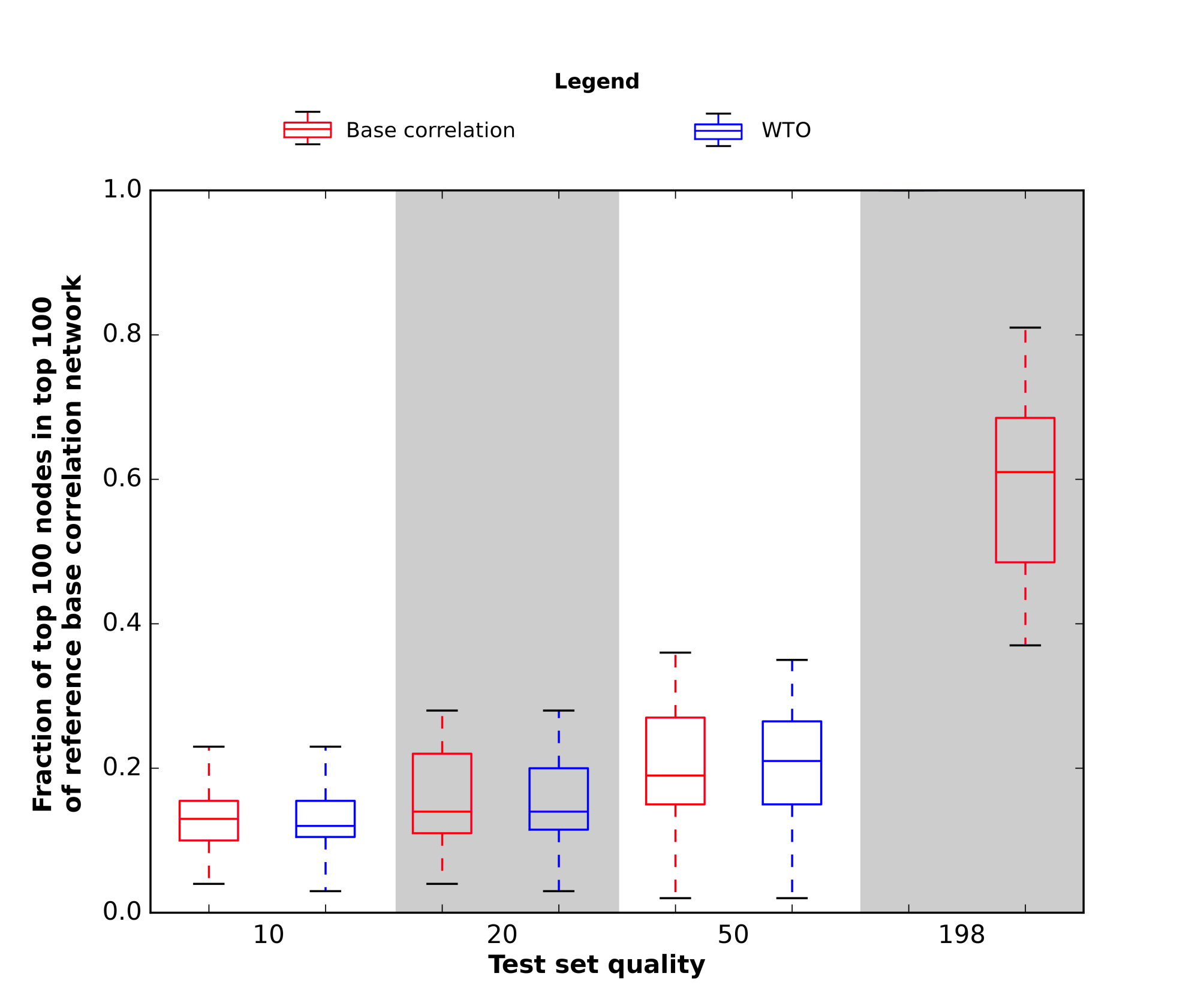

Supplement: Supplementary file 14 — Degree assortativity coefficients for networks obtained from murine brains. (PNG 107 kb) [file 12859_2019_2596_MOESM14_ESM.png]
